# Climate change and primary production: Forty years in a bunchgrass prairie

**DOI:** 10.1371/journal.pone.0243496

**Published:** 2020-12-23

**Authors:** Gary E. Belovsky, Jennifer B. Slade

**Affiliations:** 1 Environmental Research Center and Department of Biological Sciences, University of Notre Dame, Notre Dame, IN, United States of America; 2 Department of Biological Sciences, University of Notre Dame, Notre Dame, IN, United States of America; UNAM, MEXICO

## Abstract

Over the past 109 years, a Montana intermountain bunchgrass prairie annually became warmer (0.7°C) and drier (27%). The temperature and precipitation trends continued since 1978, as we studied nitrogen availability, annual aboveground primary production (ANPP), plant phenology and species composition. Given the annual increase in temperature and decrease in precipitation, ANPP might be expected to decline; however, it increased by 110%, as the period of greatest production (late-May–June) became wetter and cooler, counter to the annual pattern, and this was strongest at lower elevations. Grass production increased by 251%, while dicot production declined by 65%, which increased grass relative abundance by 54%. Summer temperatures increased 12.5% which increased plant senescence by 119% and decreased fall plant regrowth by 68%. More intense summer senescence changed plant species composition in favor of more drought tolerant species. The greater ANPP and summer senescence may increase susceptibility for fire, but fire tolerance of the plant species composition did not change. Invasive plant species increased 108% over the study with annual grasses accounting for >50% of this increase, which further increased summer plant senescence. Therefore, seasonal climate changes at a smaller geographical scale (local), rather than average annual climate changes over a larger geographical scale (regional), may better reflect plant community responses, and this makes ecological forecasting of climate change more difficult.

## Introduction

Anthropogenic induced changes in temperature and precipitation are projected to impact terrestrial primary production, e.g., [1–5]. Most of these projections focus on large spatial scales, such as biomes or across a large geographic region (>>1000 km^2^) [6], not a local ecosystem or habitat within an ecosystem (<1000 km^2^) [7–9]. In addition to climate change (e.g., temperature and precipitation), concurrently there may be increases in nitrogen availability (e.g., climate changing nitrogen mineralization, and atmospheric deposition of industrial/agricultural inputs) [10], the nutrient most often limiting terrestrial plant production. Finally, these anthropogenic changes may affect more than primary productivity, if they modify the abundances of plant species that differ in their ability to respond to changing temperature, precipitation and nitrogen regimes [11–14].

Grasslands are particularly important to humans by providing a disproportionate amount of agricultural and livestock production, as well as wildlife habitat [9]. As grasslands are often found in drier and warmer climates, they may be particularly susceptible to declining primary production and changing vegetation as climate changes [15]. Few long term data sets examine grassland responses to climate change, e.g., [8,9,16–28], and most examine large spatial scale responses to changes in annual temperature and precipitation. However, Craine et al. [23–25] indicate that seasonal shifts in temperature and precipitation may be more important at local spatial scales.

While changes in temperature and precipitation may directly affect grassland primary productivity, there may also be changes in nitrogen availability, the most common limiting nutrient. First, some studies found precipitation and nitrogen availability in grasslands to be positively correlated, which makes it difficult to distinguish between water and nitrogen limitation for primary productivity [29,30]. Second, if anthropogenic nitrogen in the atmosphere increases, then more will be deposited with increased precipitation [10]. Therefore, correlating precipitation changes with primary production may be due to water or nitrogen availability.

We examined how climate and nitrogen changes may be affecting an intermountain bunchgrass prairie. Intermountain bunchgrass prairie, one of the most endangered ecosystems in North America, once covered ~11.7 million hectares of intermountain valleys from the Rockies west to the Cascades and from southern Alberta/British Columbia south to northern Utah/Nevada, but now is restricted to less than 100,000 hectares by agriculture and overgrazing (<1% of original area) [31–34]. Bunchgrass is overlooked in grassland studies, as U.S. Great Plains and desert grassland/steppe are often the focus [8,9,35]. Bunchgrass receives much less precipitation than the Great Plains as surrounding mountains create rain shadows, and little precipitation occurs in summer (July–Sept) given prevailing Westerlies [36]. Furthermore, unlike the Great Plains, bunchgrass has a lower wildfire incidence due to less frequent lightning [36,37]. Finally, bunchgrass may be especially sensitive to climate change, as its precipitation and temperature falls within a narrow range between that for cold desert and Great Plains mixed grass prairie [32,38,39].

The bunchgrass prairie region is expected to become warmer and drier over time with anthropogenic climate change [8,9]. If drying is observed, it should lead to greater water limitation, and possibly greater nitrogen limitation, as nitrogen mineralization and atmospheric deposition may decline with less precipitation. Whether water or nitrogen is limiting, primary production should be declining over time, and drying should be increasing plant senescence over the normal summer dry period. However, vegetation responses may depend on more than annual changes over time; therefore, we also examined changes over time in seasonality that may better explain vegetation responses. In addition, climate change may affect the vegetation’s species composition, which impacts drought tolerance, fire resistance, and susceptibility to invasive species. Finally, warming and drying might convert bunchgrass prairie into cold desert steppe.

We report on a bunchgrass prairie (National Bison Range, MT, USA) with a 109-year climate record (1909–2017), 40 years of primary production measures (1978–2017), 26 years of plant composition observations (1978; 1980–1983; 1997–2017), and 24 years of soil nitrogen measures (1994–2017) for several grassland habitats.

## Study site

The National Bison Range (NBR) is one of the largest remaining tracts (~9000 ha) of intermountain bunchgrass prairie. It was never farmed and has been protected since 1908 as a U.S.F.W.S. refuge for bison (*Bison bison*). Prior to this, it was a cattle/bison ranch for less than 15 years. NBR bunchgrass prairie occurs at ~800 to 1400 m in elevation.

C_3_ grasses dominate: wheatgrasses (*Pseudoroegneria spicata*, *Pascopyrum smithii*, *Leymus cinereus*), fescues (*Festuca idahoensis*, *F*. *scabrella*), bluegrasses (*Poa pratensis*, *P*. *compressa*), junegrass (*Koleria macrantha*), and needlegrasses (*Achnatherum nelsonii*, *Hesperostipa comata*). The only C_4_ grass is three-awn (*Aristida purpurea*). Forbs include: *Achillea millefolium*, *Lupinus* spp., *Balsamorhiza sagittata*, *Heterotheca villosa*, *Taraxacum* spp., *Sympotrichum falcatum*, and *Erigeron* spp. Woody species include: *Symphoricarpus occidentalis*, *Rosa woodsii*, *Ericameria nauseosa*, *Artemisia frigida*, and *A*. *ludoviciana*. Unlike many bunchgrass areas [33,40], big sage (*A*. *tridentata*) is absent, but is in nearby valleys.

Four 2–4 ha. representative sampling sites (elevation: UTM) of NBR bunchgrass habitats (different dominant grasses) were respectively studied for 34, 27, 25 and 20 years: Hill (792m: 706453E 5249980N, *Pascopyrum smithii/Poa pratensis*); Tower 2-grassy (1380m: 708205E 5244024N, *Festuca idahoensis/Hesperostipa comata*); Trisky (1034m: 711317E 5242500N, *Festuca idahoensis/Pseudoroegneria spicata*); and Triangle (832m: 713570E 5248100N, *Pascopyrum smithii/Poa pratensis*). These four sites represent an elevation gradient and the majority of data. Four additional sites were studied for fewer years (7, 3, 3 and 1 years) and supplement the data: Hill 2 (802m: 706407E 5249831N, *Festuca idahoensis/Poa pratensis*); North Boundary (825m: 710177E 5250023N, *Pseudoroegneria spicata/Poa compressa*); Tower 2-rocky (1394m: 708212E 5243906N, *Festuca idahoensis/Pseudoroegneria spicata*); and Pauline (797m: 705717E 5247255N, *Pascopyrum smithii/Poa pratensis*). All sites were fenced to exclude large herbivore grazing (*Bison bison*, *Cervus elaphus*, *Ovis canadensis*, *Oreamnos americanus*, *Odocoileus hemionus*, *Odocoileus virginianus*, and *Antilocapra americana*).

## Methods

Field Research—permission granted by National Bison Range, Charlo, Montana - https://www.fws.gov/refuge/national_bison_range/.

### Climate

Daily climate data (precipitation, maximum and minimum temperature) are available since 1909. Data from 1909 –July, 1999 are available from the NBR headquarters (795m: 707570E 5249753N). Since July, 1999, the weather station was moved and data were not regularly collected. Therefore, data were obtained from the AgriMet Round Butte, MT station (18.9 km away, 926m: 704653E 5268696W). From 1989–2000, precipitation and temperature were highly correlated between NBR and Round Butte sites (precipitation: r = 0.94, N = 12, constant not different from 0, slope = 1.03 and p < 0.000001; temperature: r = 0.94, n = 12, constant not different from 0, slope = 0.94 and p < 0.000008).

### Nitrogen

Nitrogen available to plants was measured in 1994–2017 at four sites (Hill, Triangle, Trisky, Tower 2-grassy). N measures were obtained twice a year (warm season: June–Sept, cool season: Oct–May) using resin bags (Rexyn © Fisher Scientific) [41]. At each site, 3 bags were randomly buried 15 cm deep. Bags were kept frozen after collection until analysis by extracting N with 2M KCl, and measuring NH_4_^+^ and NO_3_^-^ with a Latchat © spectrophotometer [42]. Resin N is correlated with soil N mineralization at NBR [43].

### Vegetation

Vegetation measures included ANPP (annual aboveground net primary production), plant senescence, and plant species composition.

#### ANPP

ANPP was measured from 1978–2017. Belowground production was not measured, as it is difficult and destructive [44,45]. Two ANPP methods were employed:

1. For the period 1978–1993, live (green) vegetation was clipped on 10–12 randomly selected 0.1 m^2^ plots at each site every two weeks from late-May–mid-Oct. Clipped vegetation was separated between grass and dicot (forbs or leaves from woody vegetation), dried for 48 hours at 60°C, and then weighed. This also provided the relative abundances of grass and dicot. Clipping is time consuming and destructive, so this method was replaced.
2. Since 1994, 10–12 permanent points were randomly selected at each site, and the abundance of live (green) vegetation was measured every two weeks by radiometer [46,47] from late-May–mid-Oct. This is non-destructive and requires less time. The radiometer was held at a height to measure a 0.10 m^2^ area. Live plant biomass is computed from radiometer readings using a site and date specific regression. Each regression was based on radiometer readings of 3–5 0.1 m^2^ plots that were adjacent to the study location; these reference plots were selected to range from very low to very high plant abundances. Green vegetation in each plot was clipped, separated between grass and dicot, dried for 48 hours at 60°C, and weighed. Regressions averaged an r of 0.93 (±0.03 SE; range: 0.45–0.99). Relative abundances of grass and dicot were based on clippings from reference plots.

For either method, ANPP was the sum of increases in live (“green”) plant biomass between consecutive periods from late-May through mid-Oct. ANPP was allocated to grass and dicot using proportional composition based on clipping at each site and date (see above).

To examine whether the two different methods affected ANPP measures, clipping and radiometer measures of ANPP were both made in 1994 and found to be highly correlated (r = 0.90, n = 4) with no tendency to over- or under-estimate ANPP (slope = 0.95 ± 0.17). Furthermore, all subsequent statistical analyses were conducted with the method as an independent categorical variable and it was never significant (P always > 0.30).

#### Plant phenology

Two phenology measures were made using biweekly ANPP measurements (above): time when peak “green” biomass is attained and degree of summer senescence. Time of peak “green” biomass was determined for each site and year as the Julian day when the greatest “green” biomass was observed. Plant senescence (%) occurs over the summer (July–Aug) as “green” biomass dies or becomes dormant (“brown”) after peak “green” biomass occurs. Senescence was measured as 100 X (1 –lowest observed “green” biomass/peak observed “green” biomass).

#### Plant species composition

Plant species composition was obtained for 1978, 1980–1983 and for all years since 1997. In June 1978, vegetation was clipped and sorted to species for 26–1 m^2^ plots randomly located throughout NBR. In June 1980–1983, vegetation was clipped and sorted to species for 30–0.1 m^2^ plots randomly located at each of two sites (Hill & Triangle, which were sampled in 1978). Since June 1997, point frame sampling [43,48] was employed at the same two sites (Hill & Triangle), and all analyses were limited to these data, as earlier clipping samples were not comparable. At 18 randomly-placed permanent locations (1 m^2^) at one site and 9 at the other, 100 points in June and Sept were identified to bare ground, litter, moss, lichen, or plant species. Plant species were identified using a number of keys [49–57].

Plant nomenclature, family and life history (e.g., annual, biennial, drought resistance, fire tolerance and invasive) followed the U.S.D.A. Plant Database (http://plants.usda.gov/java/) [58]. U.S.D.A defines drought resistance and fire resistance as none, low, medium, and high. Drought resistance is based on where species grow relative to soil moisture, and fire resistance is based on regrowth and reestablishment after fire (https://plants.usda.gov › charinfo) [58]. We assigned values of 3, 2, 1 or 0 to the U.S.D.A indices (respectively, none, low, medium, and high), and computed average drought and fire resistance for the plant community (Σ[U.S.D.A’s species drought tolerance or fire resistance index X species proportion]).

### Statistics

Statistics were conducted with SYSTAT 13. Climate changes over time were analyzed using nonparametric trend analysis (Mann-Kendall rank correlation). General Linear Mixed Models (GLMM) were employed to examine whether nitrogen and vegetation measures varied among years (year as categorical independent variable) and over time during the study (year as continuous variable). Sample sites were treated as a random variable, because they were not replicated within a year. All proportions were arcsine square root transformed. Climate and elevation effects (continuous independent variables) on nitrogen and vegetation were examined using backward stepwise mixed-model regression, where independent variables were only included if P < 0.15 and the Akaike Information Criteria value (AIC) decreased.

## Results

Data for various sites at the NBR from 1978 to 2017 are presented in S1 Table (Summer Weather, Nitrogen and Vegetation) and S2 Table (Plant Species).

### Changes over time

#### Climate and plant phenology

**Fig 1:**
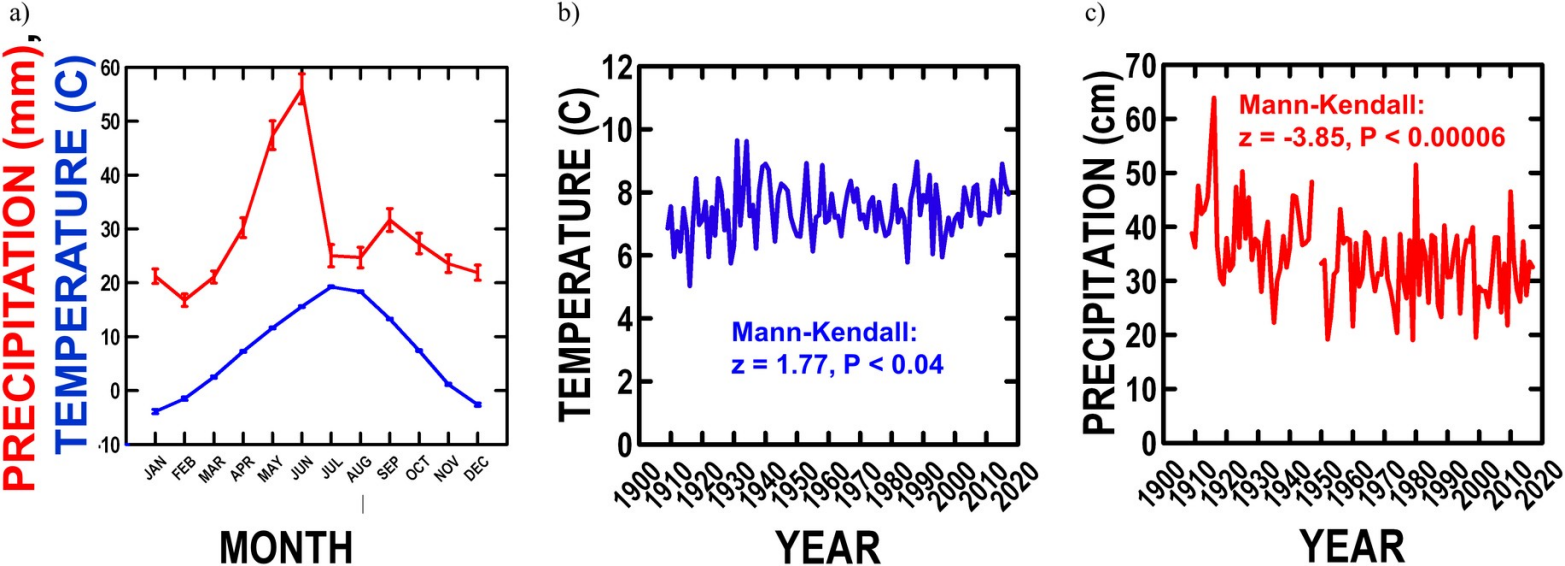
NBR climate since 1909. a) average monthly temperature and precipitation; b) trends over time in average air temperature and Mann-Kendall time series; and c) trends over time in average precipitation and Mann-Kendall time series.

Monthly average temperature and precipitation since 1909 (Fig 1A) indicate a 19.0°C maximum temperature in July–Aug, a -2.5°C minimum temperature in Dec–Jan, and a 5.7 cm maximum precipitation in May or June. As expected since 1909, average temperature increased 0.7°C (11%, Fig 1B), and average precipitation decreased 10.9 cm (27%, Fig 1C). During our study (1978–2017), average annual temperature increased (Mann-Kendall: z = 1.97, P < 0.02), which accounts for 63% of the overall temperature increase observed since 1909, so that average annual temperature increased at a 127% faster rate than before our study (0.018°C/yr vs. 0.008°C/yr). Likewise, annual precipitation decreased during our study (Mann-Kendall: z = -1.99, P < 0.02), which accounts for 21% of the overall precipitation decrease since 1909, so that average annual precipitation decreased by a 68% slower rate than before our study (0.19 cm/yr vs. 0.06 cm/yr).

**Fig 2:**
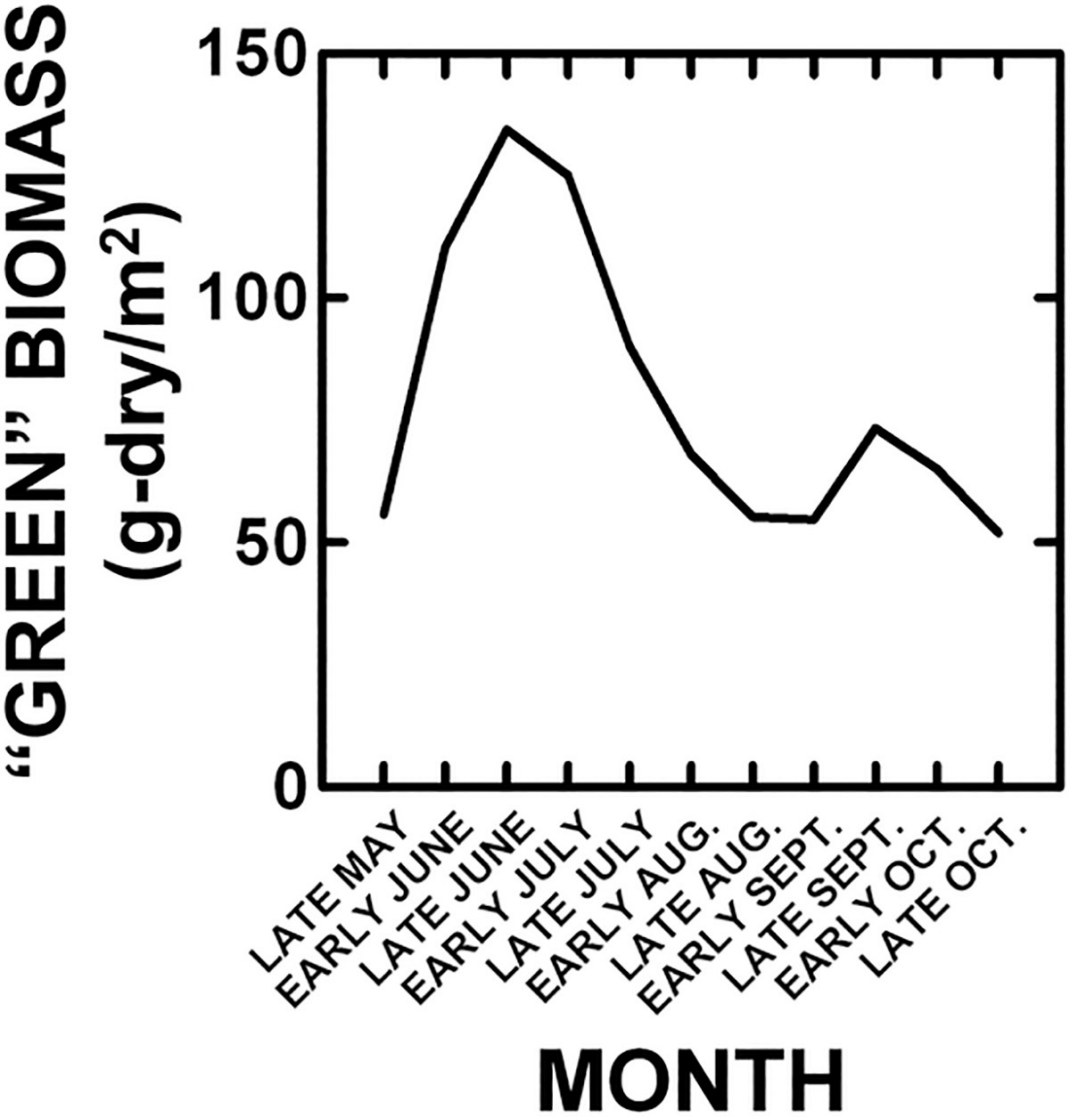
Average monthly “green” plant biomass from the Hill site as an example.

Seasonal values for precipitation and temperature trends during our study were also examined (Table 1). Like the annual trends, Water Year (Oct–Sep) precipitation tended to decrease and temperature to increase. Seasonally, precipitation tended to decrease in spring, summer and recharge period (winter + spring), increase in fall and remain unchanged in winter. Seasonally, temperature tended to increase primarily in summer, and remain unchanged in other seasons. Because the majority (> 88%) of plant growth (peak “green” biomass) occurs at NBR from late-May–June (Fig 2), the decrease in Water Year, recharge period and spring, like annual, precipitation suggests that annual plant production should decline over our study.

**Table 1:** Seasonal precipitation and temperature trends during our study (1978–2017) based on Mann-Kendall trend series.

	PRECIPITATION:		TEMPERATURE:	
	Direction & Mann-Kendall z	P	Direction & Mann-Kendall z	P
**Water Year (prev. Oct–current Sept)**	-1.22	0.11	+1.21	0.11
**Winter (Jan–Mar)**	+0.47	0.32	+0.27	0.39
**Spring (Apr–Jun)**	-1.41	0.08	-0.65	0.74
**Summer (Jul–Sept)**	-2.33	0.01	+3.54	0.0002
**Fall (Oct–Dec)**	+3.04	0.001	+0.48	0.31
**Recharge (winter + spring)**	-0.51	0.30	0.00	0.50

Climate changes at more specific times during vegetation phenology were examined. During the main period of plant growth (late-May–June: Fig 2), temperature tended to remain unchanged (Mann-Kendall: z = -0.52, P < 0.30), as found for spring in general, while precipitation tended to increase (Mann-Kendall: z = 1.23, P < 0.11), opposite the spring seasonal value. Therefore, the ratio of precipitation to temperature tended to increase (Mann-Kendall: z = 1.11, P < 0.13) over the period of main plant growth during our study. This indicates reduced evapotranspiration [59,60], which is counter to the annual, Water Year, recharge period, and spring seasonal drying and general warming, and suggests increased annual plant production.

There is a period of summer plant senescence (July–Aug decline in “green” plant biomass: Fig 2), as the vegetation is dominated by “cool-season” grasses (C_3_: >>95% of bomass). During this period, temperature increased (Mann-Kendall: z = 3.37, P < 0.0004) and precipitation decreased (Mann-Kendall: z = -2.24, P < 0.01), which are consistent with the summer seasonal trends. This led to a decline in the precipitation to temperature ratio (Mann-Kendall: z = -2.60, P < 0.005), as expected with annual and summer seasonal drying and warming. This suggests that senescence should have intensified over our study.

There is a brief period when plants grow again (late-Sept increase in “green” biomass: Fig 2) after summer senescence and before the onset of frosts (Oct). During this period, temperature increased (Mann-Kendall: z = 2.20, p < 0.02) as expected for the summer season, and precipitation was unchanged (Mann-Kendall: z = -0.86, p < 0.20), which is opposite the summer seasonal decrease and fall seasonal increase. This tended to reduce the precipitation to temperature ratio (Mann-Kendall: z = -1.11, p < 0.13), which should have decreased this renewal of plant production during our study.

NBR climate patterns are similar (r = 0.92–0.96) to observations at two nearby weather stations (1989–1999 Round Butte: 18.9 km away, elevation = 926m, 704653E 5268696N, and 1978–1999 St. Ignatius: 13.2 km away. Elevation = 897m, 719607E 5244815N).

#### Nitrogen available to plants

**Fig 3:**
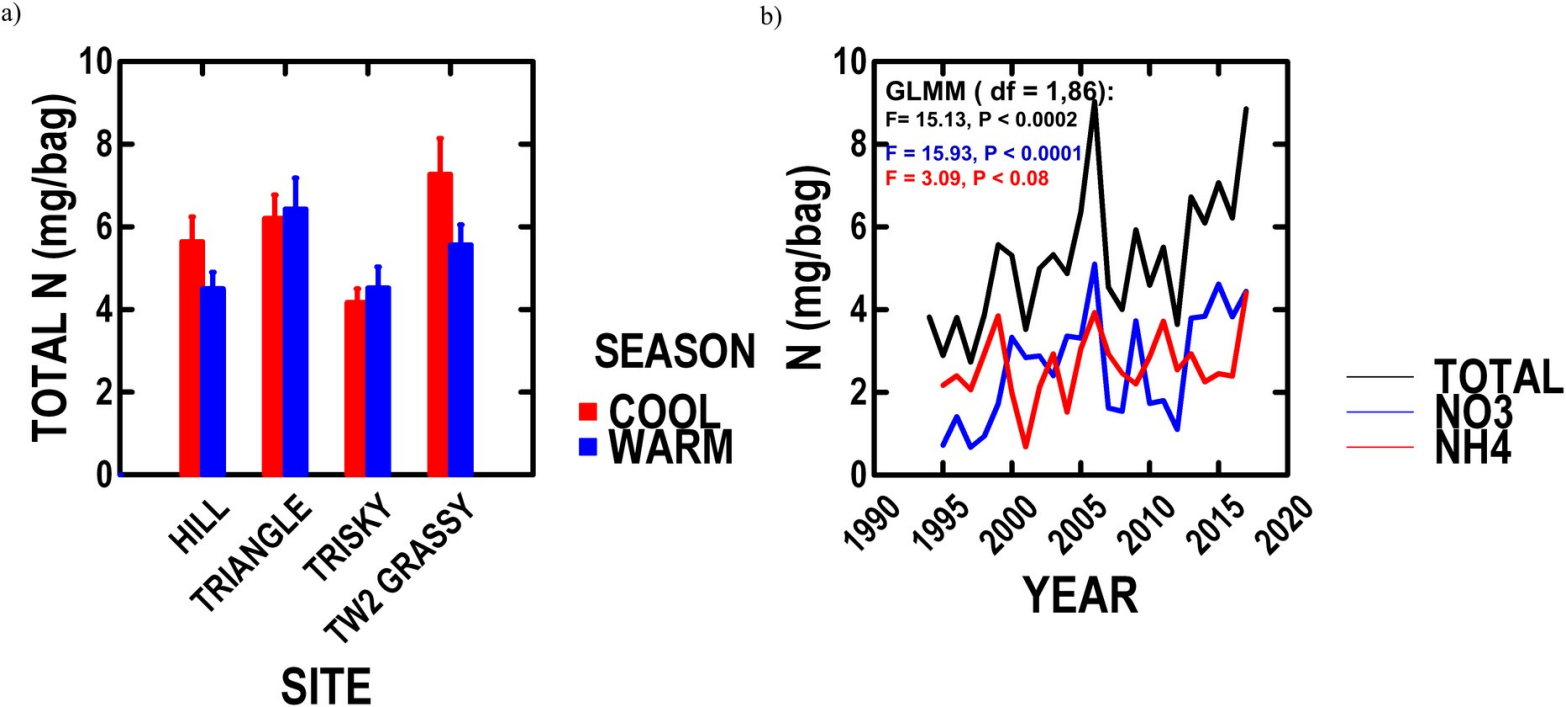
NBR soil nitrogen since 1995 for four sample sites studied longest. a) average cool (Oct–May) and warm (June–Sept) season soil total nitrogen at each site, and b) trends over time for warm season soil NO_3_^-^, NH_4_^+^, and total nitrogen across sites with GLMM results.

Total nitrogen (NO_3_^-^ + NH_4_^+^) was not different between cool (Oct–May) and warm (June–Sept) seasons (Fig 3A: t = -1.14, P < 0.26). Total nitrogen tended to vary among years (1995–2017, Table 2: P < 0.09), especially for the warm season (Table 2: P < 0.02). Total nitrogen tended to exhibit an increasing trend over our study (Table 2: P < 0.09), especially for the warm season (Table 2: P < 0.0002). Cool season NO_3_^-^, NH_4_^+^ and total nitrogen exhibited no trends over our study (respective GLMM: P < 0.38, 0.31, 0.42), but warm season NO_3_^-^, NH_4_^+^ and total nitrogen increased over our study (Fig 3B) respectively by 200.8%, 38.6% and 94.8%.

**Table 2:** GLMM results for soil, vegetation and plant community measurements among years and trend over the years of our study (- = decline, 0 = no change, + = increase). Significant (p < 0.05) results are in bold.

	AMONG YEARS				OVER YEARS		
	df	F	P	Trend	df	F	P
**TOTAL NITROGEN -**							
Cool season	22,65	1.58	0.08	0	1.83	0.80	0.37
Warm season	23,67	1.80	**0.03**	**+**	1,86	15.13	**0.0002**
Annual	22,65	1.54	0.09	0	1,83	2.86	0.09
**VEGETATION -**							
ANPP	38,90	4.25	**0.000001**	**+**	1,127	37.1	**0.000001**
Peak biomass—day	32,77	2.41	**0.00009**	**-**	1,108	27.1	**0.000001**
% Grass	38,90	5.58	**0.000001**	**+**	1,127	45.6	**0.000001**
Senescence	31,77	3.37	**0.000008**	**+**	1,107	54.1**2**	**0.000001**
**PLANT COMMUNITY -**							
**COMMUNITY -**							
Diversity	20,16	1.31	0.29	+	1,35	7.45	**0.01**
Richness	20,16	3.76	**0.005**	**+**	1,35	5.33	**0.03**
Bluegrasses	20,16	10.4	**0.000009**	**-**	1,35	50.1	**0.000001**
Wheat grasses	20,16	8.91	**0.00002**	**+**	1,35	23.8	**0.00002**
**PLANT CHARACTERISTICS**							
** CHARACTERS -**							
Drought resistance	20,16	2.35	**0.04**	**+**	1,35	23.8	**0.00002**
Fire tolerance	20,16	1.20	0.36	-	1,35	3.30	0.08
Invasives (%)	20,16	5.45	**0.0006**	**+**	1,35	4.77	**0.04**

#### Vegetation

**Fig 4:**
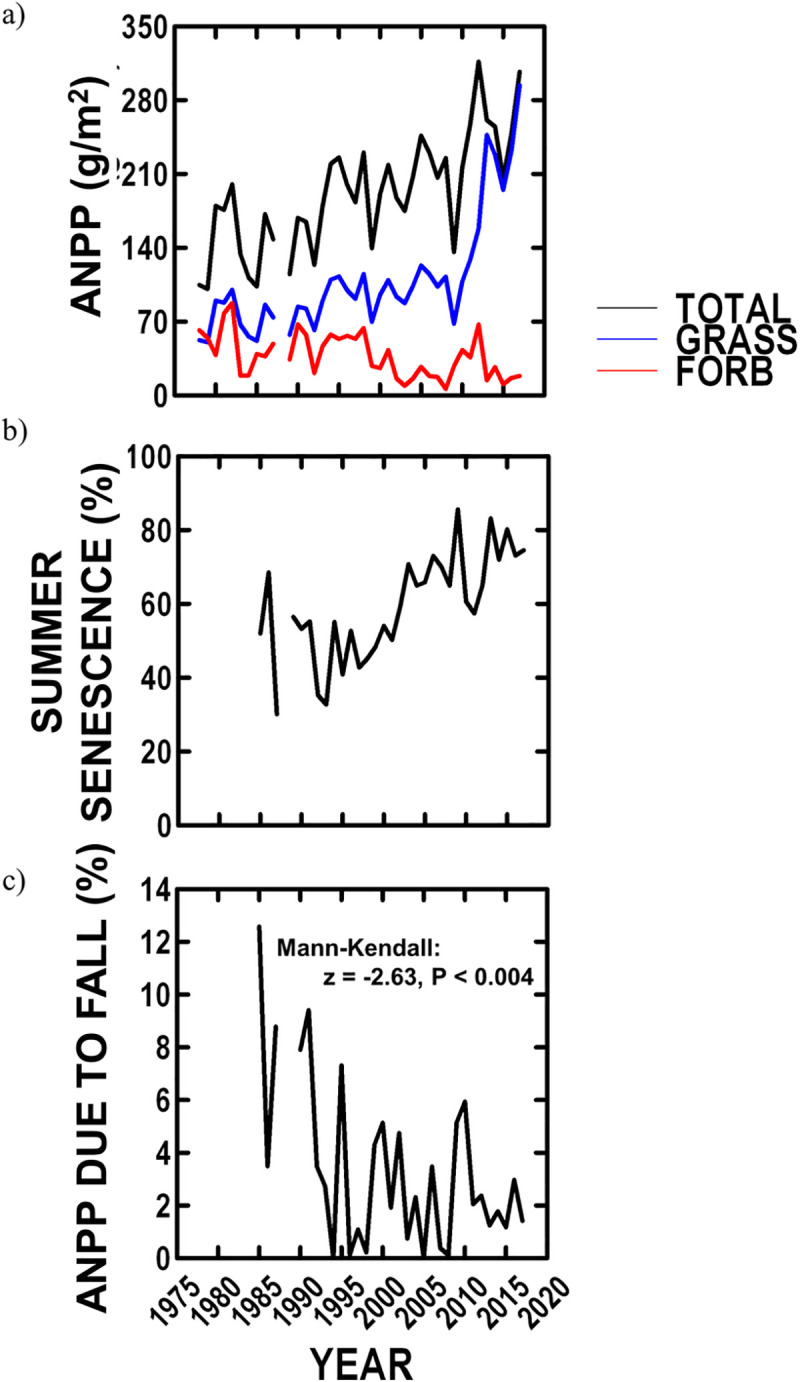
NBR vegetation since 1978 averaged across all sites in a year. a) trend over time in ANPP for grass, forb and in total; b) trend over time in summer percent plant senescence; and c) trend over time in fall plant production (% ANPP).

Over our study, ANPP averaged 191.5 (± 8.6 SE) g/m^2^ (among years: 100.9–316.4 g/m^2^) (Fig 4A), and significantly varied among years and increased by 110.4% over our study (Table 2, Fig 4A). Grass accounted on average for 79.2% (± 2.2 SE) (among years: 41.0–97.3%) of ANPP (Fig 4A). Grass production increased by 251%, while forb production decreased by 65% over our study (Fig 4A). Consequently, grass relative abundance increased by 54% from 61% to 96% (Table 2, P < 0.000001) of ANPP over our study. Finally, the time (Julian day) at which peak “green” biomass (>88% of ANPP) was observed (late-May–June: Fig 2) varied among years and declined by 23 days (16%) from 1985–2017 (Table 2).

Summer plant senescence averaged 59.2% (± 2.5 SE) (among years: 30.1–85.6%) (Fig 4B), significantly varied among years and increased 119% over our study (Table 2, Fig 4B). Renewed fall plant production after the period of summer senescence (Fig 2) accounted on average for 3.5% of ANPP (± 0.6 SE) (among years: 0–12.6%), and significantly decreased by 67.8% over our study (Fig 4C).

**Fig 5:**
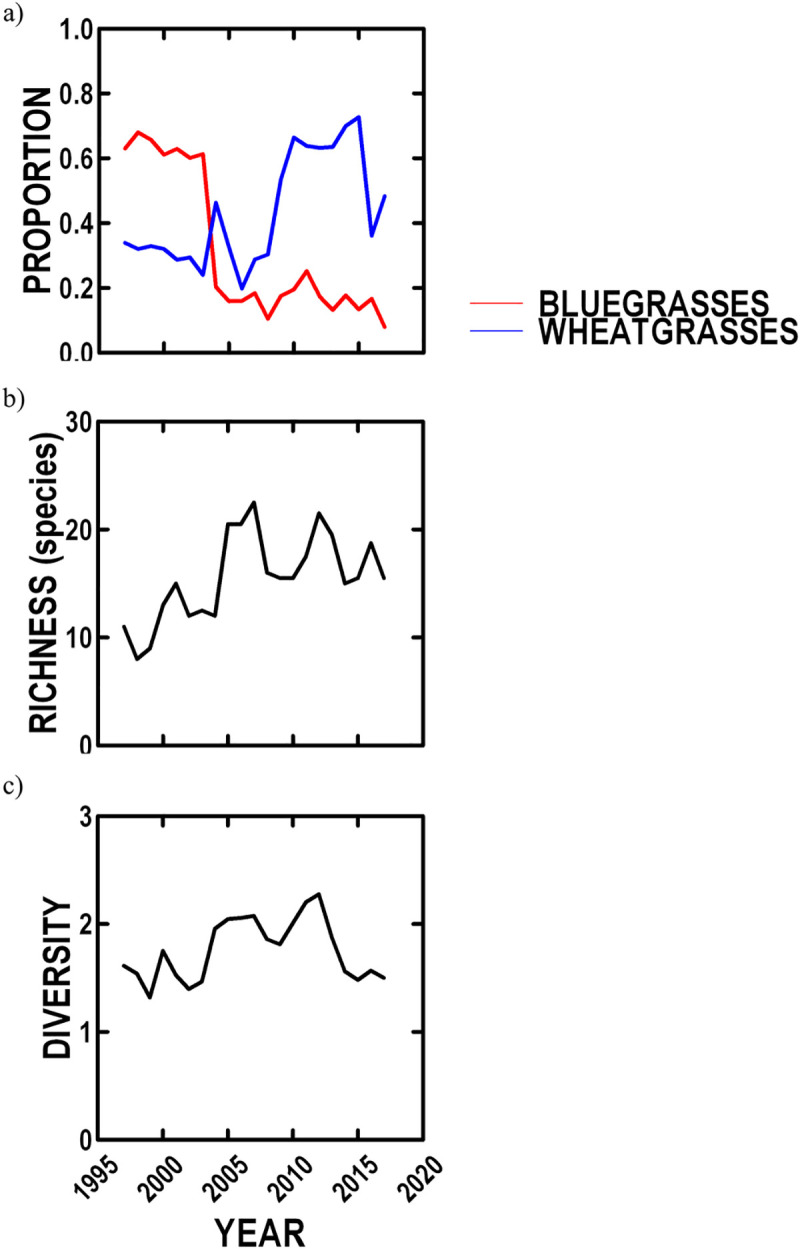
NBR plant species composition based on point frame incidence since 1997 for two sites. a) trends over time in percentage of grass composition for bluegrasses and wheatgrasses; b) trends over time in plant species richness; c) trends over time in plant Shannon diversity.

Plant species composition was only measured repeatedly at two sites (Hill, Triangle: 2001–2017) with the same methodology (point frame). Because grasses are the dominant species, we examined the relative abundances of the two most common types of grass: bluegrasses and wheatgrasses, which varied in relative abundance among years (Table 2, Fig 5A). Bluegrasses declined by 89% and wheatgrasses increased by 143% over our study (Table 2, Fig 5A). Species richness based on all species significantly varied among years and increased by 51.7% over our study (Table 2, Fig 5B) with rare dicots accounting for the increase (P < 0.0001) as grass species did not change (P < 0.54) and increased in abundance. Shannon diversity did not vary among years, but increased by 26.5% over our study (Table 2, Fig 5C).

**Fig 6:**
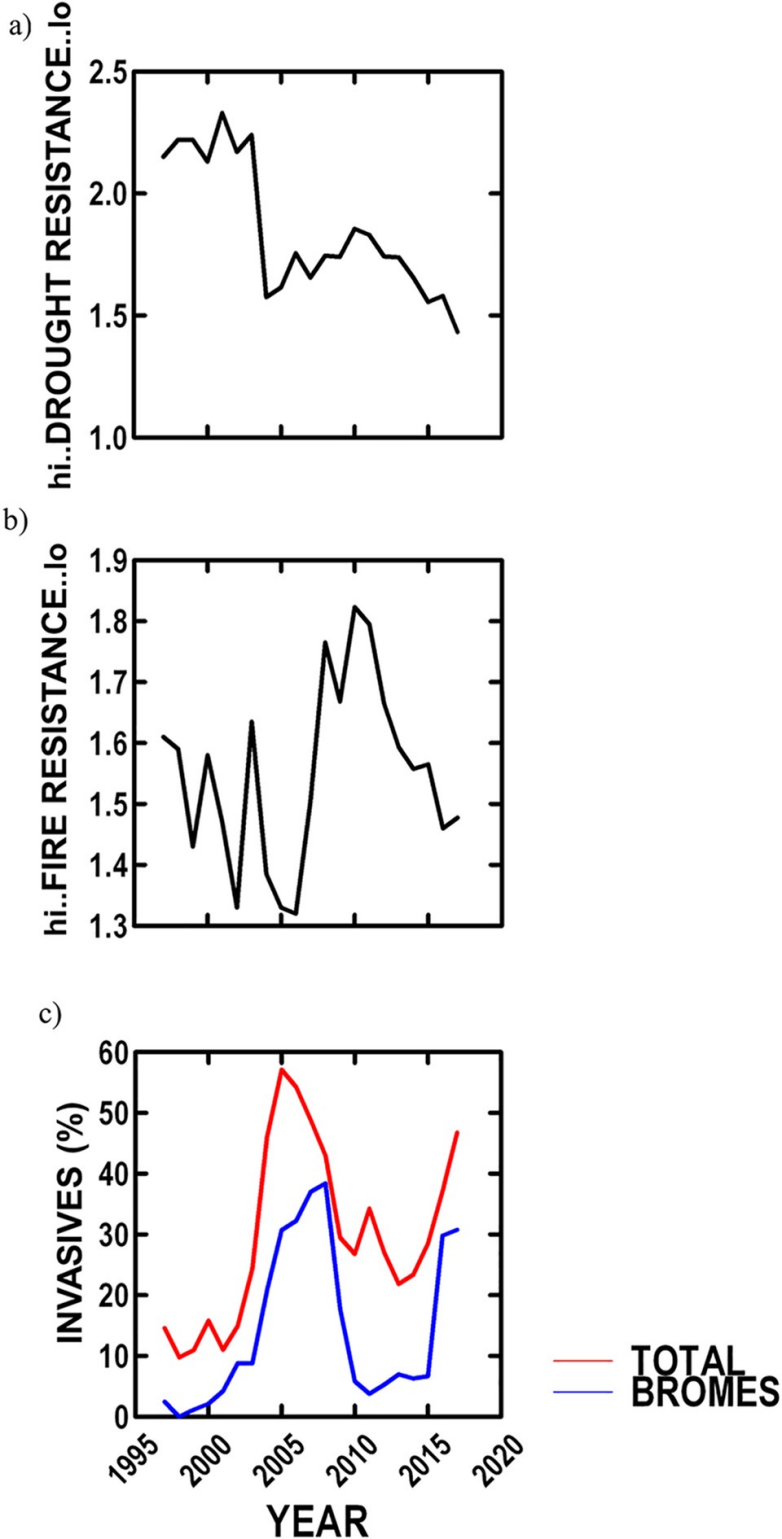
NBR plant species characteristics based on the USDA plant database and point frame incidence since 1997 at two sites. a) annual trends in average plant drought resistance; b) annual trends in average plant fire tolerance; and c) annual trends in invasive plant incidence, in total and annual brome grasses.

Plant characteristics based on the USDA plant database (drought resistance, fire tolerance, and invasive species) at the two sample sites were examined. Drought resistance significantly varied among years, and increased 35.8% over our study (Table 2, Fig 6A). Fire tolerance did not vary among years, and tended to decrease over our study (Table 2, Fig 6B). Incidence of invasive species (average = 29.8%) increased by 108% over our study (Table 2, Fig 6C). The increase in invasives was largely due to non-native annual brome grasses, which comprised on average 50% of invasives. Number of invasive grass species increased from 1 to 3, and invasive dicots increased from 3 to 10 species.

### Environmental correlates

#### Nitrogen availability

Warm and cool season total nitrogen were not correlated with prior season’s total nitrogen (respectively, P < 0.92 and 0.88), suggesting that nitrogen does not accumulate. Summer (June–July) temperature and sample site elevation were positively correlated with warm season total nitrogen (Table 3).

**Table 3:** Backward stepwise regression results, where variables are included if their P < 0.15 and the AIC with them declines.

	SIGNS	r	N	P	AIC
**Warm season (June–Sept) N -**					
summer temperature	+	0.30	94	0.004	452.78
summer temperature, elevation	+,+	0.36	94	0.003	450.28
**ANPP -**					
June precipitation/temperature	+	0.32	136	0.0001	1512.24
June prec/temp, prev. ANPP	+,+	0.62	125	0.000001	1348.19
June prec/temp, prev. ANPP, elevation	+,+,+	0.63	125	0.000001	1346.63
**Grass ANPP -**					
Previous year grass ANPP	+	0.70	125	0.000001	1327.37
Previous year grass ANPP, June prec/temp	+,+	0.73	125	0.000001	1318.86
**Forb ANPP -**					
Previous year forb ANPP	+	0.44	125	0.000001	1165.12
Previous year forb ANPP, grass ANPP	+,-	0.47	125	0.000001	1163.51
Previous year forb ANPP, grass ANPP, elevation	+,-,+	0.51	125	0.000001	1159.17
Previous year forb ANPP, grass ANPP, elevation, June prec/temp	+,-,+,+	0.52	125	0.000001	1158.91
**Peak biomass—day**					
% grass	-	0.27	112	0.004	977.10
June prec/temp	+	0.35	112	0.008	972.94
**Senescence -**					
Jul–Aug temperature	+	0.51	112	0.000001	-75.76
Jul–Aug temperature, grass ANPP	+,+	0.53	112	0.000001	-76.95
**Fall ANPP -**					
Jul—Aug temperature	-	0.31	32	0.07	-57.71

#### Vegetation

Environmental trends (precipitation, temperature, nitrogen) were compared with vegetation trends.

#### ANPP

Increasing ANPP over our study was unexpected, because typically employed precipitation and temperature trends (annual, Water Year and water recharge period) along with the absence of an increase in annual nitrogen availability suggest decreasing ANPP. Considering that the majority (>88%) of ANPP occurred in late-May–June (the growing season), again the spring seasonal precipitation declined and temperature and cool season nitrogen remained unchanged. However, within the spring season, precipitation increased and temperature remained unchanged for the late-May–June period, so the ratio of precipitation to temperature (evapotranspiration) increased, which is consistent with the observed increasing ANPP. As a result, the observed late-May–June ratio of precipitation to temperature was positively correlated with ANPP (Table 3). In addition, ANPP was also affected by factors that did not trend over our study (Table 3). First, ANPP was greater with higher ANPP in the prior year. Second, sampling sites were selected to provide an elevation gradient, and ANPP increased with elevation. Finally, the Julian day at peak “green” biomass was negatively correlated with percent of ANPP comprised of grass and positively correlated with late-May–June ratio of precipitation to temperature (Table 3).

#### % Grass

Grass ANPP relationships are the same as for total ANPP, but forb ANPP responded somewhat differently (Table 3). While forb ANPP responded positively with prior year forb ANPP and elevation, it only weakly increased with the ratio of June precipitation to temperature, and current year grass ANPP had a strong negative effect, indicating potential competition. Therefore, while grass ANPP benefitted by the changing environmental conditions, forbs did not and were reduced by the increasing grasses.

#### Senescence

Plant senescence in summer increased with summer temperature and the abundance of grass (Table 3). Summer precipitation, the summer ratio of precipitation to temperature, and elevation had no effect on senescence.

#### Fall ANPP

Fall plant production increased as summer temperature decreased, i.e., less summer senescence (Table 3). Fall production was not related to total ANPP (negative correlation, P < 0.13), indicating it is independent of the primary growing season (late-May–June) production.

#### Plant community

Plant species composition was affected by summer senescence, as bluegrasses decreased (P < 0.000007) and wheatgrasses increased (P < 0.05) with summer senescence. Diversity indices were unaffected by summer senescence (P < 0.48), while species richness increased with summer senescence (P < 0.04).

#### Plant characteristics

Drought resistance and relative abundance of invasive species increased as summer senescence increased (respectively, P < 0.0005 and 0.01). Fire tolerance was not related to any variables.

## Discussion

The once abundant intermountain bunchgrass prairie has not been well studied. Most past bunchgrass studies focused on plant community composition, e.g., [33,40,61].

### Vegetation

#### ANPP changes

The few estimates of bunchgrass prairie ANPP range from 50–230 g-dry/m^2^, e.g., [33,62–65], did not track ANPP changes among years or with environmental variables, or annual phenology. We found a much more variable ANPP (78–395 g-dry/m^2^) at NBR with variation among sampling sites within a year from 40–60% and variation among years within a site from 110–260%. NBR ANPP is known to increase with experimental addition of water and nitrogen fertilizer [43,60,66–70]. We observed ANPP to be positively correlated with the growing season (late-May–June) ratio of precipitation to temperature and with elevation. Elevation should be positively correlated with precipitation and negatively correlated with temperature, and we also observed it to be positively related to nitrogen availability. ANPP also increased with the previous year’s ANPP. Therefore, moisture (growing season ratio of precipitation to temperature) appears to be more important to ANPP than nitrogen.

First, late-May–June (main period of growth: > 88% of ANPP) ratio of precipitation to temperature reflects moisture available for plant growth as evaporation increases at higher temperatures, so more precipitation is needed to achieve a given level of plant growth [59,60]. This ratio was experimentally demonstrated to correlate with evapotranspiration and ANPP [60]. Second, nitrogen availability from natural dynamics [29,30] and anthropogenic deposition [10,30] has been shown to increase with precipitation in grasslands. We did not observe this as warm season total nitrogen was not correlated with precipitation, but increased with summer temperature, which reflects rapid mineralization [71,72]. Also, NBR is in an area of low atmospheric deposition of anthropogenic nitrogen [73]. Finally, previous year’s ANPP was positively correlated with current ANPP, which indicates that moisture and nitrogen (elevation) have greater impact when plants already are abundant after a year of good growth probably due to a greater plant stem density after a year of higher growth [27,74].

Most striking, NBR ANPP increased by 110% over our 40-year study as the ratio of late-May–June (main period of plant growth: > 88% of ANPP) precipitation to temperature increased due to wetter and cooler conditions. Grass ANPP over our study increased by 251%, while dicot ANPP declined by 65%, leading to a 54% increase in grass relative abundance. The driving factor again was specific seasonal, not annual, climate change (wetter and cooler late-May–June) favoring the “cool-season” (C_3_) grasses that are most abundant (> 99%). Furthermore, the increase in these grasses appeared to reduce forb abundance through competition, as the grasses form clumps (cespitose) that are spaced apart to avoid competition for water, as denoted by the prairie being called “bunchgrass”. Finally, ANPP changes were stronger at low elevations over our study, which may be expected as higher elevations are generally moister and cooler to begin with than low elevations.

Our ANPP observations are counter to expectation given that NBR has become warmer and drier based on annual averages over the past 109 years, as reported elsewhere for western Montana [7,75]. This result indicates that specific seasonal, not annual, climate change trends may be more important [8,13,23–25] at local spatial scales [11–14], and questions U.S. grassland projections made at large spatial scales (national and regional) based on annual average climate values [8,9,76,77].

#### Phenology changes

The Julian day on which peak “green” plant biomass (> 88% of ANPP) occurs declined by 23 days (16%) over our study. This large phenological change was not related to increasing temperature as often projected [7,75], but related to increasing grass relative abundance and the increasing ratio of late-May–June precipitation to temperature. As noted earlier, NBR grasses are almost exclusively (> 99%) “cool season” (C_3_), which grow rapidly and more abundantly with wetter and cooler conditions. Therefore, the peak period of growth may be more affected by changing species composition and specific seasonal, not annual, climate trends. At another western Montana location, earlier flowering dates for dicots are reported, but this is correlated with different local seasonal climate trends [78] than presented here.

Summer senescence (% decline in “green” plant biomass from the peak) increased by 119% over our study, which correlated with increasing summer temperature and grass ANPP. Again, specific seasonal, not annual, climate change was more important as summer (July–Aug) temperature increased more than the average annual value (2.3°C vs. 0.7°C). Furthermore, senescence is expected to be more intense for the most abundant “cool season” grasses. Finally, increased senescence had no apparent effect on ANPP, as ANPP is largely determined by the ratio of late-May–June precipitation to temperature supporting “cool season” grass growth.

Fall re-initiation of plant growth declined by 67.8% over our study, which correlated with increasing summer temperature, i.e., greater summer senescence. Fall plant production now is very low in most years, as it may be more difficult for plants to overcome severe summer senescence. This differs from the historic pattern of fall “greening” in this ecosystem [33]. For example, in 1805, Lewis and Clark mentioned this [79], and 19^th^ and early 20^th^ century settlers [80–82] relied on this to provide livestock forage after summer senescence.

#### Plant community and characteristic changes

Bluegrasses decreased by 89% and wheatgrasses increased by 143% over our study, as summer senescence intensified. The majority of bluegrass and wheatgrass species in our study are natives that form clumps (cespitose) and can spread vegetatively (rhizomatous). However, bluegrasses are more shallowly rooted than wheatgrasses (~25 vs. 40 cm) and would be more negatively affected by warm and dry conditions during summer (less drought tolerant, USDA plant database). The dramatic post-2005 increase in wheatgrasses may be due to a steady rise in summer temperatures (0.05°C/yr: P < 0.0003) and senescence (>1%/yr: P < 0.03) until 2005 and then summer temperature (21.3°C: P < 0.45) and senescence (29%: P < 0.21) stabilize.

Species richness increased by 51.7% over our study, as summer senescence intensified. Species richness is important in grasslands as it strongly affects ecosystem functioning [83], which has been experimentally demonstrated [84]. Cleland et al.’s [85] survey of grassland regions found that richness increased with low precipitation, but we found that it increased with summer temperature, which may be explained if regional low precipitation and high summer temperature are positively correlated. However, while richness increased, the Shannon diversity index, which includes richness and evenness of abundance, only weakly increased (26.5%) over our study, which is counter to expectation [85]. Therefore, greater richness must be countered by less evenness, if more drought resistant species are added and become more common, while less drought resistant species are not lost, but become rarer.

Richness and diversity may in part have increased as the incidence of invasive species increased by 108%, as summer senescence intensified, which is consistent with observations that grassland invasives increase with drying conditions [86]. Increasing invasives had no effect on NBR ANPP (P < 0.28), and thereby, would have little effect on summer senescence measures, as > 50% of invasives were annual brome grasses, which provide very little biomass (cheatgrass, *Bromus tectorum*, accounted for less than 3% of the invasive bromes). Average plant drought resistance increased by 35.8% over our study, which is largely due to the increase in wheatgrasses and decrease in bluegrasses. Fire tolerance was unchanged over our study.

### Future of bunchgrass prairie

Seasonal, rather than annual, changes in climate and their strong directional effect on the NBR vegetation, may be affecting the small areas of remaining bunchgrass prairie elsewhere. Climate change can be directional or part of a natural cycle. Using time series partial autocorrelation and Fourier analysis on the 109 year NBR climate record, one can observe 4–6 and 10–15 year cycles in precipitation and temperature, as expected, given cyclic oceanic drivers [87–89]. These cycles cannot account for the 40-year directional changes observed, but can only modify them [89,90]; rather an unidentified cycle with periodicity greater than twice our 40-year study period would be needed to produce directional changes, which seems unlikely. Therefore, a likely explanation for our observations is anthropogenic climate change [7,75,87,91–93].

The identified seasonal climate changes also may impact intermountain bunchgrass prairies in other ways, including increased invasive species impacts and susceptibility to fire. Invasives are dramatically increasing at other intermountain bunchgrass prairies [94]. Invasives do not influence NBR ANPP, because most are small annual plants that do not add much biomass (>50% are annual grasses). However, these annual plants contribute to increased summer senescence and thereby, may intensify the potential for fire.

Historically, intermountain bunchgrass had low fire incidence compared to Great Plains grassland due to less frequent lightning [36,37], but higher summer temperatures spawn more lightning [95–97], and summer temperature is increasing in the bunchgrass. Therefore, greater ANPP and summer senescence in NBR bunchgrass provides more fuel for increased lightning to spawn fires than in the past. Furthermore, we found that plant species that are more resistant to fire are not increasing in abundance, further enhancing the potential for fire disturbance.

What the intermountain bunchgrass will look like in the future given climate changes and associated disturbances is uncertain? There is very little difference between our study valley with bunchgrass prairie (Mission Valley, MT) and an adjacent valley (Little Bitterroot Valley, MT) (respectively, average annual precipitation of 40.5 vs. 37.5 cm and annual temperature of 7.4 vs. 7.5°C) [98]. There is no evidence that the less productive cold desert steppe [99] will replace the bunchgrass, as steppe is dominated by shrubs and we find that shrubs are declining (P < 0.004, N = 35) along with other dicots. This is counter to Harte et al. [14] reporting that woody vegetation is replacing Colorado grassland with observed and experimental warming. If the observed increase in NBR grass ANPP and relative abundance continues, then historic bunchgrass ecosystem may morph into a different, possibly previously unknown, type of grassland. This uncertainty is consistent with claims that local and seasonal climate change may create very different ecosystems [8,13,23–25].

## Conclusion

Climate over the past 109 years in a bunchgrass ecosystem has annually warmed and dried as expected with anthropogenic climate change. Over the past 40 years, we found that changes in plant production, composition and summer senescence followed seasonal, not annual, climate trends. The period of greatest (> 88%) plant production (late-May–June) became wetter and cooler counter to annual trends, which resulted in increased plant production, especially for grasses. Summer temperature became warmer than expected from annual trends, which resulted in increased summer senescence and decreased fall regrowth. Therefore, our data support claims that ecological forecasts for the consequences of climate change should be based on local [11–14] and seasonal [8,13,23–25] climate change, not regional and annual values.

The above and additional patterns (e.g., nitrogen changes, species composition, invasive species, and possible increased fire) reported here indicate that intermountain bunchgrass prairie is no longer the historic ecosystem, and may be morphing into a previously unknown ecosystem. Therefore, ecologists may have difficulty in forecasting how ecosystems will respond to climate change and other associated disturbances without far greater ecological knowledge at local and seasonal levels as we describe here.

## Supporting information

S1 Table Summer weather, nitrogen and vegetation. Summer weather, nitrogen and vegetation data are provided from 1978 to 2017 at various sites on the National Bison Range (NBR).
(XLSX)

S2 Table Plant species. Plant species data are provided for various sites on the National Bison Range (NBR) from 1978–2017.
(XLSX)
